# The classification of obesity based on metabolic status redefines the readmission of non-Hodgkin’s lymphoma—an observational study

**DOI:** 10.1186/s40170-023-00327-x

**Published:** 2023-12-06

**Authors:** Hang Dong, Honglin Guo, Jing Du, Yiping Cheng, Dawei Wang, Junming Han, Zinuo Yuan, Zhenyu Yao, Ran An, Xiaoqin Wu, Kyle L. Poulsen, Zhixiang Wang, Shanshan Shao, Xiude Fan, Zhen Wang, Jiajun Zhao

**Affiliations:** 1 Department of Endocrinology, Shandong Provincial Hospital, Shandong University, Jinan, Shandong 250021 China; 2 Key Laboratory of Endocrine Glucose & Lipids Metabolism and Brain Aging, Ministry of Education; Department of Endocrinology, Shandong Provincial Hospital Affiliated to Shandong First Medical University, Jinan, Shandong 250021 China; 3 Department of Endocrinology, The First Affiliated Hospital of Baotou Medical College, Baotou, 014010 China; 4 Shandong Clinical Research Center of Diabetes and Metabolic Diseases, Jinan, Shandong 250021 China; 5 Shandong Institute of Endocrine and Metabolic Diseases, Jinan, Shandong 250021 China; 6 “Chuangxin China” Innovation Base of stem cell and Gene Therapy for endocrine Metabolic diseases, Jinan, Shandong 250021 China; 7 Shandong Engineering Laboratory of Prevention and Control for Endocrine and Metabolic Diseases, Jinan, Shandong 250021 China; 8 Department of Mechanical and Aerospace Engineering, Shandong Engineering Research Center of Stem Cell and Gene Therapy for Endocrine and Metabolic Diseases, Jinan, Shandong 250021 China; 9 Department of Mechanical and Aerospace Engineering, Case Western Reserve University, Cleveland, OH USA; 10 Department of Integrative Biology and Pharmacology, McGovern Medical School at UTHealth, Houston, TX USA; 11 Department of Anesthesiology, McGovern Medical School, University of Texas Health Science Center at Houston, Houston TX, USA

**Keywords:** Non-Hodgkin lymphoma, Readmission, Obesity, Metabolic abnormality, Phenotype

## Abstract

**Background:**

The relationship between obesity and non-Hodgkin’s lymphoma (NHL) was controversial, which may be due to the crudeness definition of obesity based on body mass index (BMI). As obesity and metabolic abnormalities often coexist, we aimed to explore whether the classification of obesity based on metabolic status can help to evaluate the real impact of obesity on the readmission of NHL.

**Methods:**

In this retrospective cohort study, utilizing the 2018 Nationwide Readmissions Database, we identified NHL-related index hospitalizations and followed them for non-elective readmission. The patients with NHL were classified as metabolically healthy non-obese (MHNO) and obese (MHO) and metabolically unhealthy non-obese (MUNO) and obese (MUO). Readmission rates for each phenotype were calculated at 30-day intervals. Multiple COX regression was used to analyze the association of metabolic-defined obesity with 30-day, 90-day, and 180-day readmission rates in patients with NHL.

**Results:**

There were 22,086 index hospitalizations with NHL included. In the multivariate COX regression, MUNO was associated with increased 30-day (HR = 1.113, 95% CI 1.036–1.195), 90-day (HR = 1.148, 95% CI 1.087–1.213), and 180-day readmission rates (HR = 1.132, 95% CI 1.077–1.189), and MUO was associated with increased 30-day (HR=1.219, 95% CI: 1.081-1.374), 90-day (HR = 1.228, 95% CI 1.118–1.348), and 180-day readmission rates (HR = 1.223, 95% CI 1.124–1.33), while MHO had no associations with readmission rates.

**Conclusions:**

The presence of metabolic abnormalities with or without obesity increased the risk of non-selective readmission in patients with NHL. However, obesity alone had no associations with the risk of non-selective readmission, suggesting that interventions for metabolic abnormalities may be more important in reducing readmissions of NHL patients.

**Supplementary Information:**

The online version contains supplementary material available at 10.1186/s40170-023-00327-x.

## Introduction

Non-Hodgkin lymphoma (NHL) is associated with an increasing burden globally, reaching 509,590 cases and 248,724 deaths in 2018 [1], which is closely related to considerable economic burden and resource utilization. According to the National Cancer Institute in the USA, national expenditures of the USA for the NHL were nearly $18.6 billion in 2020 [2]. Therefore, it is necessary to alleviate the huge financial burden of patients with non-Hodgkin lymphoma, and reducing the hospital readmission rate may be one of the effective measures. Among patients discharged from the hospital, nearly one in five were readmitted within 30 days, and 34.0% within 90 days for all causes [3]. It is reported that rehospitalizations accounted for about $26 billion for Medicare patients alone a year in the USA [4]. Most readmissions are non-elective, related to the deterioration of the patient’s clinical condition, and it harms the welfare of patients and increases the medical burden [5]. Thus, it is urgent to reduce non-elective readmission in patients with NHL, especially by improving risk factors that can be modified.

Obesity, a vast public health problem, was demonstrated to track closely with increased morbidity and mortality rates among various types of cancers [6, 7]. In the USA, 39.8% of the population was obese in 2015–2016 [8] and it is projected that obesity will affect more than half of the population by 2030 [9]. As an adjustable factor, it is important to explore whether weight loss can improve cancer outcomes. However, the association of obesity with the prognosis of NHL was highly controversial. Many studies reported that obesity led to poor outcomes in patients with NHL [10, 11], while some studies reported exactly the opposite observation [12]. In addition, some studies showed that obesity had no associations with the prognosis of NHL [13]. The crudeness of BMI as a measure of obesity may be an important reason [14]. Obesity is closely associated with metabolic syndrome components, including diabetes, hypertension, and hyperlipidemia [15]. It is reported that more than a third of the population has metabolic syndrome in the USA [16], which has been proven to have adverse effects on the risk and prognosis of NHL in studies [17–19]. However, BMI-based obesity always classifies people with different metabolic statuses into the same category, which may lead to confused research conclusions. Metabolic-defined obesity was presented and may be a solution to the above questions. Metabolically healthy obesity was proved to be associated with lower content of ectopic fat (visceral and liver), better cardiorespiratory fitness, and maintenance of insulin sensitivity and beta cell function [20]; and it was reported that metabolically healthy obesity was associated with a lower risk of some adverse health outcomes, including major adverse cardiovascular events [15], type 2 diabetes [21], and depression [22], compared with metabolically unhealthy obesity. Metabolic-defined obesity may help to better clarify the real effect of obesity on the prognosis of NHL. Therefore, it is necessary to explore the role of metabolic-defined obesity in patients with NHL to guide assessment and improvement of prognosis.

Utilizing the Nationwide Readmissions Database (NRD), which is a powerful database designed to support analyses of national readmission, we aimed to analyze the association of metabolic-defined obesity with non-elective readmission among patients with NHL, to better elucidate the real association between obesity and NHL and alleviate the readmission of patients with NHL.

## Methods

### Data source

To investigate the association of metabolic-defined obesity with readmission in patients with NHL, we conducted this retrospective cohort study. The study population was extracted from the United States 2018 NRD database developed as part of the Healthcare Cost Utilization Project (HCUP) by the Agency for Healthcare Research and Quality (AHRQ) [23]. The NRD is the largest publicly available all-payer inpatient care database in the USA, which is designed to support various types of analyses of national readmissions. It contained data from 28 geographically dispersed states, accounting for 59.7% of the total USA resident population and 58.7% of all USA hospitalizations. The sampling frame for the NRD was limited to discharges for patients treated at community hospitals in the NRD States that were not rehabilitation or long-term acute care facilities. All of the discharges in the sampling frame were included. Patients were identified utilizing a reliable, verified linkage number, enabling tracking hospitalized patients within a participating state during an individual year, while following strict privacy guidelines. Because of the publicly availability and de-identified character of the NRD, our study did not require Institutional Review Board approval.

### Study population

Using International Classification of Diseases, Tenth Edition (ICD-10) diagnosis codes, we identified NHL-related index hospitalizations in 2018. The subtypes of NHL according to the ICD-10 codes were shown in the Supplement Table S1. We excluded (1) patients who had a diagnosis of being underweight (*n* = 1837), other malignant tumors(*n* = 4531), or pregnancy (*n* = 43); (2) patients who died at index hospitalization(*n* = 2055), (3) patients with missing data (*n* = 656). To ensure 180-day follow-up, patients discharged after July were excluded (*n* = 18926), as admissions could not be tracked across years in the NRD. Figure S1 and Table S2 in the Supplement showed the Flowchart of cohort selection and ICD-10 codes used in inclusion and exclusion respectively.

### Definitions of body weight and metabolic status

Obesity was considered as a BMI ≥ 25 kg/m^2^. Metabolic abnormalities (hyperglycemia, hypertension, hyperlipidemia) were determined by hospitalization diagnosis according to the availability of data. ICD-10 codes for obesity and metabolic abnormalities are shown in Table S2 in the Supplement. A person was considered “metabolically unhealthy” with two or more kinds of metabolic abnormalities [24]. Participants were grouped based on their obesity categories (obese or non-obese) and metabolic status (healthy or unhealthy), which resulted in four categories: metabolically healthy non-obese (MHNO), metabolically healthy obese (MHO), metabolically unhealthy non-obese (MUNO) and metabolically unhealthy obese (MUO).

To better elucidate the role of obesity and metabolic abnormality, we used the two following categories: (1) people were mutually exclusively categorized into 10 groups: non-obese patients with no metabolic abnormality, non-obese patients only with hyperglycemia, non-obese patients only with hypertension, non-obese patients only with hyperlipidemia, and non-obese patients with multiple metabolic abnormalities; obese patients were also divided into five groups; (2) people were categorized according to weight status and number of metabolic abnormality components, which resulted in eight groups: no metabolic abnormality and non-obese, one metabolic abnormality and non-obese, two metabolic abnormalities and non-obese, three metabolic abnormalities and non-obese; obese patients were also divided into four similar groups.

### Outcomes measured

Our outcome of interest was the risk for 30-day readmission, 90-day readmission, and 180-day readmission. The definition of the index hospitalization was the first NHL-related hospitalization during a specific calendar year. After the index admission was determined, subsequent readmissions of the corresponding patients in the same calendar year were tracked. If a patient had multiple readmissions within a year, only the first readmission was included in the analysis. Hospital transfers, trauma-related readmissions, and elective hospital readmissions, such as hospitalizations to maintain chemotherapy were excluded to better study the readmissions associated with changes in condition. The time interval between the readmissions and the index admissions was calculated to determine whether readmissions were within 30 days, 90 days, and 180 days. In addition, we extracted the main diagnosis at readmission based on ICD-10 codes, to show the main cause of readmission (Supplement Table S3).

### Data analysis

Continuous variables were represented as mean ± standard deviation (SD) and compared using the one-way analysis of variance test. Categorical variables were shown as frequency (percentage) and compared using the Pearson chi-square test. Kaplan-Meier curves were plotted and differences in readmission were tested by log-rank test. COX regression analysis was used to assess readmission, adjusted for age and sex in Model 1. Further adjustments for discharge disposition, discharge month, local hospital admission, household income, emergency department service, insurance type, and location of residence were conducted as Model 2, and these factors were described in detail in the supplement Text S1. Comorbidities in the Royal College of Surgeons’ adaptation of the Charlson comorbidity index (CCI) was extracted, and the number of comorbidities was further adjusted as a categorical variable (0, 1, 2, or ≥ 3) in Model 3 [25]. ICD-10 codes for CCI were given in Table S4 in the Supplement. A stratified analysis based on sex subgroup was conducted. We also analyzed the association of metabolic-defined obesity with aggressive NHL. ICD-10 codes for aggressive NHL and related results were presented in the Supplement (Table S5, Text S2, and Figure S2). Statistically significant was defined as *p* < 0.05 and all tests were 2-tailed. All analysis was performed in SPSS 26.0 (IBM SPSS Inc., Chicago, USA).

## Results

### Baseline characteristics

We identified a total of 22086 NHL-related index hospitalizations from January through June 2018. As shown in Table 1, a majority of these patients (74.9%) were older than 60 years at index admission, and the proportion of males (57.2%) was higher than females (42.8%). Patients with chronic lymphocytic leukemia of B-cell type or diffuse large B cell lymphoma accounted for the highest proportion, both above 20%. (Supplement Table S1). In total, 19374 (87.7%) were non-obese (MHNO: *n* = 12367[56.0%]; MUNO: *n* = 7007[31.7%]), and 2712 (12.3%) were obese (MUO: *n* = 1294 [5.9%]; MUO: *n* = 1418[6.4%]). Compared with metabolically healthy patients, patients who were metabolically unhealthy were more concentrated in the elderly group. The proportion of patients older than 60 years was higher among metabolically unhealthy patients than metabolically healthy patients, both in non-obese (90.8% vs 67%) and obese patients (80.3% vs 58.9%).

**Table 1:** Baseline characteristics of the patients and hospital by obesity defined by metabolic status

Variables	All	Non-Obesity		Obesity		*P*
		Metabolically healthy *n* = 12367(56.0)	Metabolically unhealthy *n* = 7007(31.7)	Metabolically healthy *n* = 1294(5.9)	Metabolically unhealthy *n* = 1418(6.4)	
Age						< 0.001
≤ 44	1918 (8.7)	1627 (13.2)	78 (1.1)	187 (14.5)	26 (1.8)	
45–59	3623 (16.4)	2455 (19.9)	569 (8.1)	345 (26.7)	254 (17.9)	
60–74	8329 (37.7)	4453 (36.0)	2643 (37.7)	504 (38.9)	729 (51.4)	
≥ 75	8216 (37.2)	3832 (31.0)	3717 (53.0)	258 (19.9)	409 (28.8)	
Male	12639 (57.2)	6991 (56.5)	4181 (59.7)	698 (53.9)	769 (54.2)	< 0.001
Discharge disposition						< 0.001
Routine	13814 (62.5)	8378 (67.7)	3809 (54.4)	835 (64.5)	792 (55.9)	
Care facility	3548 (16.1)	1663 (13.4)	1416 (20.2)	188 (14.5)	281 (19.8)	
Home with home health care	4549 (20.6)	2204 (17.8)	1746 (24.9)	265 (20.5)	334 (23.6)	
Against medical advice	164 (0.7)	116 (0.9)	32 (0.5)	5 (0.4)	11 (0.8)	
Discharged alive but destination unknown	11 (0.0)	6 (0.0)	4 (0.1)	1 (0.1)	0 (0.0)	
Hospitalization at a local hospital	1485 (6.7)	881 (7.1)	414 (5.9)	95 (7.3)	95 (6.7)	0.01
Household income						< 0.001
0-25th percentile	4714 (21.3)	2596 (21.0)	1446 (20.6)	327 (25.3)	345 (24.3)	
26th to 50th percentile	5815 (26.3)	3188 (25.8)	1870 (26.7)	346 (26.7)	411 (29.0)	
51st to 75th percentile	5764 (26.1)	3222 (26.1)	1817 (25.9)	357 (27.6)	368 (26.0)	
76th to 100th percentile	5793 (26.2)	3361 (27.2)	1874 (26.7)	264 (20.4)	294 (20.7)	
Emergency department service	14298 (64.7)	7397 (59.8)	5062 (72.2)	824 (63.7)	1015 (71.6)	< 0·001
Primary expected payer						< 0·001
Medicare	14120 (63.9)	6859 (55.5)	5639 (80.5)	642 (49.6)	980 (69.1)	
Medicaid	1759 (8.0)	1280 (10.4)	246 (3.5)	151 (11.7)	82 (5.8)	
Private insurance	5377 (24.3)	3686 (29.8)	953 (13.6)	444 (34.3)	294 (20.7)	
Self-pay	310 (1.4)	232 (1.9)	36 (0.5)	21 (1.6)	21 (1.5)	
Other	520 (2.4)	310 (2.5)	133 (1.9)	36 (2.8)	41 (2.9)	
Patient location						<0·001
“Central” counties of metro areas of >= 1 million population	6157 (27.9)	3525 (28.5)	1904 (27.2)	374 (28.9)	354 (25.0)	
“Fringe” counties of metro areas of >= 1 million population	6233 (28.2)	3412 (27.6)	2098 (29.9)	336 (26.0)	387 (27.3)	
Counties in metro areas of 250,000–999,999 population	4715 (21.3)	2636 (21.3)	1512 (21.6)	276 (21.3)	291 (20.5)	
Counties in metro areas of 50,000–249,999 population	2025 (9.2)	1121 (9.1)	618 (8.8)	128 (9.9)	158 (11.1)	
Micropolitan counties	1667 (7.5)	946 (7.6)	480 (6.9)	107 (8.3)	134 (9.4)	
Not metropolitan or micropolitan counties	1289 (5.8)	727 (5.9)	395 (5.6)	73 (5.6)	94 (6.6)	
Charlson comorbidity index						<0·001
0	9507 (43.0)	6440 (52.1)	2059 (29.4)	596 (46.1)	412 (29.1)	
1	6725 (30.4)	3697 (29.9)	2183 (31.2)	404 (31.2)	441 (31.1)	
2	3600 (16.3)	1506 (12.2)	1568 (22.4)	205 (15.8)	321 (22.6)	
>= 3	2254 (10.2)	724 (5.9)	1197 (17.1)	89 (6.9)	244 (17.2)	

Among non-obese individuals, those who were metabolically unhealthy were less likely to be discharged routinely (54.4% vs 67.7%) but more likely to transfer to a care facility (20.2% vs 13.4%), or accept home health care (24.9% vs 17.8%) after discharge. We observed a similar pattern for metabolically unhealthy versus metabolically healthy patients among obese subjects. This may mean that metabolically unhealthy patients with NHL were likely to have poorer recovery at discharge, both in non-obese and obese patients.

Metabolically unhealthy patients were more likely to have multiple comorbidities, both in obese and non-obese patients. In non-obese patients, more than half of the metabolically healthy patients had no comorbidities, whereas only 29.4% of the metabolically unhealthy patients did. Similarly, the proportion of patients without comorbidities in MHO was much higher than in MUO patients (46.1% vs 29.1%). The proportion of patients with more than three comorbidities was higher among metabolically unhealthy patients than metabolically healthy patients, in both non-obese patients (17.1% vs 5.9%) and obese patients (17.2% vs 6.9%).

### Readmission rate

As shown in Supplement Figure S3, most patients (19.3%) with NHL were readmitted 30 days after the index hospitalization. As the period increased, the readmission rate decreased gradually. By 180 days after discharge, nearly 40% of patients have been readmitted to the hospital. Supplement Table S3 presented the main diagnosis of the readmission. About 40% of patients were readmitted to the hospital for NHL. The proportions of patients readmitted to hospital for infections or parasitic diseases, circulatory system diseases and respiratory diseases were also high, all at around 7 to 8%.

### Metabolic abnormalities and obesity

**Fig. 1:**
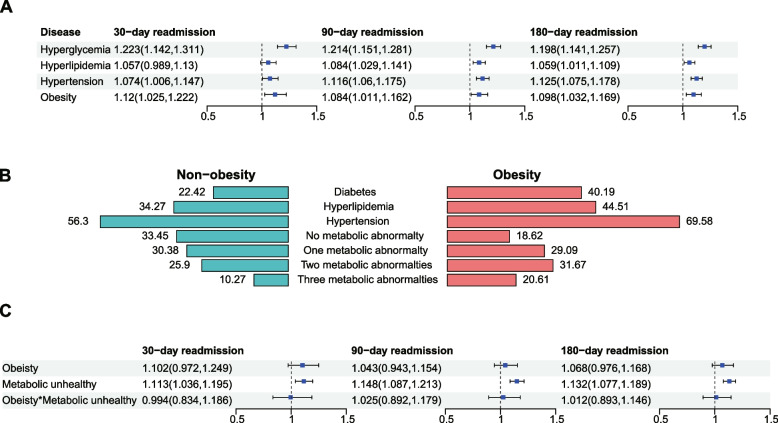
The association of metabolic abnormalities and obesity with readmission of patients with NHL. **A** The association of metabolic abnormalities and obesity with readmission of patients with NHL. Factors adjusted in the COX regression: age, sex, discharge disposition, discharge month, local hospital admission, household income, emergency department service, insurance type, and location of residence. **B** The proportion of metabolic abnormalities in obese and non-obese patients. **C** Impact of metabolic abnormalities on the association between obesity and readmission of patients with NHL. Obesity, metabolic abnormalities, and their interaction item were included in COX regression analysis, adjusted for age, sex, discharge disposition, discharge month, local hospital admission, household income, emergency department service, insurance type, and location of residence

Hyperglycemia, hyperlipidemia, hypertension, and obesity were all associated with a higher risk of readmission of NHL patients in COX regression. However, it is worth noting that obese patients had a higher incidence of metabolic abnormalities, including hyperglycemia, hyperlipidemia, and hyperlipidemia, than non-obese patients (Fig. 1B). Furthermore, COX regression showed that although there was no interaction between obesity and metabolic abnormalities, the association between obesity and readmission disappeared after adjustment for metabolic abnormalities (Fig. 1C). Therefore, it is necessary to systematically assess the association of obesity and metabolic abnormalities with the readmission of NHL patients by incorporating metabolic-defined obesity.

### Metabolic-defined obesity

**Fig. 2:**
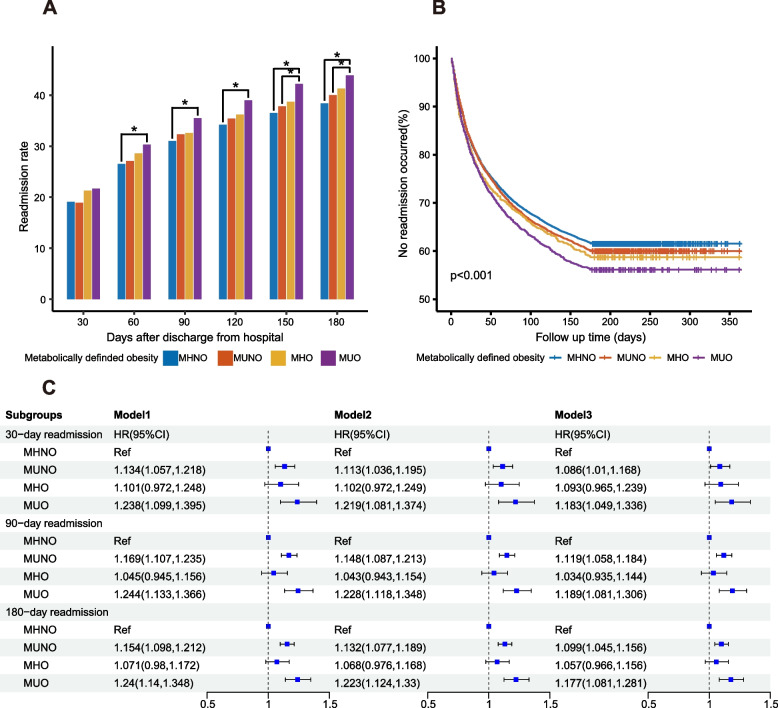
The association of obesity defined by metabolic status with readmission of patients with NHL. **A** Readmission rates by the number of days following discharge from index hospitalization, stratified by metabolically defined obesity. The symbol * meant that the readmission rates of the two groups were significantly different at the 0.05 level after the Bonferroni correction. **B** Kaplan-Meier curve for readmission of NHL patients, stratified by metabolically defined obesity. **C** COX analysis was conducted to assess the association between metabolically defined obesity status and NHL readmission. Factors adjusted in the COX regression: Model1: age, sex; Model2: Model1+ discharge disposition, discharge month, local hospital admission, household income, emergency department service, insurance type, and location of residence; Model3: Model2+ CCI. Abbreviations: MHNO, metabolically healthy non-obese; MUNO, metabolically unhealthy non-obese; MHO, metabolically healthy obese; MUO, metabolically unhealthy obese

When the discharge time was longer than 60 days, readmission rates were higher in MUO than in MHNO. Readmission rates of 150 and 180 days were higher for MUO than for MUNO (Fig. 2A). The survival curve showed that compared with MHNO, both MUNO and MHO had a higher readmission rate, while MUO had the highest readmission rate. The log-rank test suggested a significant difference in readmission rates between different groups (*P* < 0.001) (Fig. 2B).

In COX regression, after adjustment for sex and age (Model 1), metabolic ill-health was associated with higher readmission risk at 30, 90, and 180 days in both obese and non-obese patients, compared with MHNO (Fig. 2C). There were no associations between MHO and 30-day, 90-day, and 180-day readmissions. In Model 2, after further adjustment for other confounding factors, MUNO was associated with higher 30-day (HR = 1.113, 95% CI 1.036–1.195), 90-day (HR = 1.148, 95% CI 1.087–1.213), and 180-day readmission rates (HR = 1.132, 95% CI 1.077–1.189), MUO was associated with higher 30-day (HR = 1.219, 95% CI 1.081–1.374), 90-day (HR = 1.228, 95% CI 1.118–1.348), and 180-day readmission rates (HR = 1.223, 95% CI 1.124–1.33), while MHO was not associated with readmission rate. These results suggested that metabolic ill-health was a risk factor for readmission in both obese and non-obese people, while obesity alone had no associations with readmission. Further adjustment for CCI showed the same results in Model 3.

### Obesity defined by a single metabolic abnormality

Non-obese patients only with hyperglycemia demonstrated a greater risk of 30-day (HR = 1.367, 95% CI 1.147–1.629), 90-day (HR = 1.295, 95% CI 1.124–1.492), and 180-day readmission (HR = 1.184, 95% CI 1.038–1.351) than non-obese patients with no metabolic abnormality (Table 2). Non-obese patients only with hypertension had a high risk of 180-day readmission (HR = 1.07, 95% CI 1.002–1.142). Obese patients only with hypertension had a greater risk of 30-day (HR = 1.243, 95% CI 1.03–1.501), 90-day (HR = 1.168, 95% CI 1.003-1.36), and 180-day readmission (HR = 1.235, 95% CI 1.081–1.411). Non-obese patients only with hyperlipidemia had an increased risk of 30-day readmission (HR = 1.163, 95% CI 1.001–1.353).

**Table 2:** The association of obesity defined by a single metabolic abnormality with readmission

Subgroups	30-day readmission		90-day readmission		180-day readmission	
	HR (95% CI)	*P* value	HR (95% CI)	*P* value	HR (95% CI)	*P* value
Non-obesity without metabolic abnormality	1(reference)		1(reference)		1(reference)	
Non-obesity with hyperglycemia	**1.367(1.147,1.629)**	**< 0.001**	**1.295(1.124,1.492)**	**< 0.001**	**1.184(1.038,1.351)**	**0.012**
Non-obesity with hyperlipidemia	**1.163(1.001,1.353)**	**0.049**	1.108(0.982,1.249)	0.095	1.09(0.978,1.213)	0.119
Non-obesity with hypertension	1.033(0.94,1.135)	0.505	1.062(0.987,1.143)	0.108	**1.07(1.002,1.142)**	**0.042**
Non-obesity with multiple metabolic abnormalities	**1.167(1.071,1.271)**	**< 0.001**	**1.206(1.128,1.289)**	**< 0.001**	**1.185(1.116,1.257)**	**< 0.001**
Obesity without metabolic abnormality	1.05(0.86,1.282)	0.632	1.022(0.871,1.198)	0.792	0.998(0.864,1.154)	0.983
Obesity with hyperglycemia	1.341(0.932,1.927)	0.114	1.221(0.905,1.647)	0.192	1.185(0.902,1.557)	0.222
Obesity with hyperlipidemia	0.937(0.602,1.458)	0.774	0.875(0.613,1.248)	0.46	0.962(0.712,1.301)	0.803
Obesity with hypertension	**1.243(1.03,1.501)**	**0.023**	**1.168(1.003,1.36)**	**0.046**	**1.235(1.081,1.411)**	**0.002**
Obesity with multiple metabolic abnormalities	**1.274(1.121,1.447)**	**< 0.001**	**1.285(1.163,1.42)**	**< 0.001**	**1.275(1.166,1.395)**	**< 0.001**

COX analysis was conducted to assess the association of obesity defined by a single metabolic abnormality with readmission, adjusted for age, sex, discharge disposition, discharge month, local hospital admission, household income, emergency department service, insurance type, and location of residence

### Obesity defined by the number of metabolic abnormality components

Among non-obese patients, one, two, and three metabolic abnormalities were all associated with an increased risk of readmission of 30, 90, and 180 days (Table 3). Compared with non-obese patients without metabolic abnormalities, non-obese patients with one, or two metabolic abnormalities had respectively an approximately 0.1-fold and 0.15-fold increased risk of readmission, while the risk of readmission increased by about 0.3 times among non-obese patients with three metabolic abnormalities. These results indicated that the risk of readmission increased with the number of comorbid metabolic abnormalities, for both short-term and long-term readmission. For obese patients, compared with non-obese patients without metabolic abnormality, obesity with one metabolic abnormality was associated with an increased risk of 30-day(HR = 1.213, 95% CI 1.032–1.426) and 180-day readmission (HR = 1.184, 95% CI 1.056–1.328), obesity with two metabolic abnormalities was associated with an increased risk of readmission of 30 days (HR = 1.326, 95% CI 1.137–1.546), 90 days (HR = 1.315, 95% CI 1.164–1.485), and 180 days (HR = 1.301, 95% CI 1.166–1.451), and obesity with three metabolic abnormalities was associated with an increased risk of 90-day (HR = 1.242, 95% CI 1.071–1.441), and 180-day readmission (HR = 1.238, 95% CI 1.083–1.414). In obese patients, the HR value for readmission risk was lower in patients with three metabolic abnormalities than those with two metabolic abnormalities. One possible reason was that the risk of death after discharge from the hospital may increase in obese patients with three metabolic abnormalities, which may further affect their readmission rate.

**Table 3:** The association of obesity defined by the number of metabolic abnormality components with readmission

Subgroups	30-day readmission		90-day readmission		180-day readmission	
	HR (95% CI)	*P* value	HR (95% CI)	*P* value	HR (95% CI)	*P* value
Non-obesity metabolic healthy	1(reference)		1(reference)		1(reference)	
Non-obesity with one metabolic abnormality	**1.091(1.002,1.188)**	**0.044**	**1.094(1.024,1.17)**	**0.008**	**1.085(1.022,1.152)**	**0.007**
Non-obesity with two metabolic abnormalities	**1.123(1.023,1.233)**	**0.014**	**1.162(1.081,1.249)**	**< 0.001**	**1.145(1.073,1.221)**	**< 0.001**
Non-obesity with three metabolic abnormalities	**1.281(1.139,1.441)**	**< 0.001**	**1.321(1.207,1.447)**	**< 0.001**	**1.29(1.188,1.399)**	**< 0.001**
Obesity metabolic healthy	1.05(0.86,1.282)	0.63	1.022(0.871,1.198)	0.791	0.999(0.864,1.154)	0.984
Obesity with one metabolic abnormality	**1.213(1.032,1.426)**	**0.019**	1.131(0.993,1.289)	0.064	**1.184(1.056,1.328)**	**0.004**
Obesity with two metabolic abnormalities	**1.326(1.137,1.546)**	**< 0.001**	**1.315(1.164,1.485)**	**< 0.001**	**1.301(1.166,1.451)**	**< 0.001**
Obesity with three metabolic abnormalities	1.198(0.988,1.454)	0.067	**1.242(1.071,1.441)**	**0.004**	**1.238(1.083,1.414)**	**0.002**

COX analysis was conducted to assess the association of obesity defined by the number of metabolic abnormalities with readmission, adjusted for age, sex, discharge disposition, discharge month, local hospital admission, household income, emergency department service, insurance type, and location of residence

### Sex differences

**Fig. 3:**
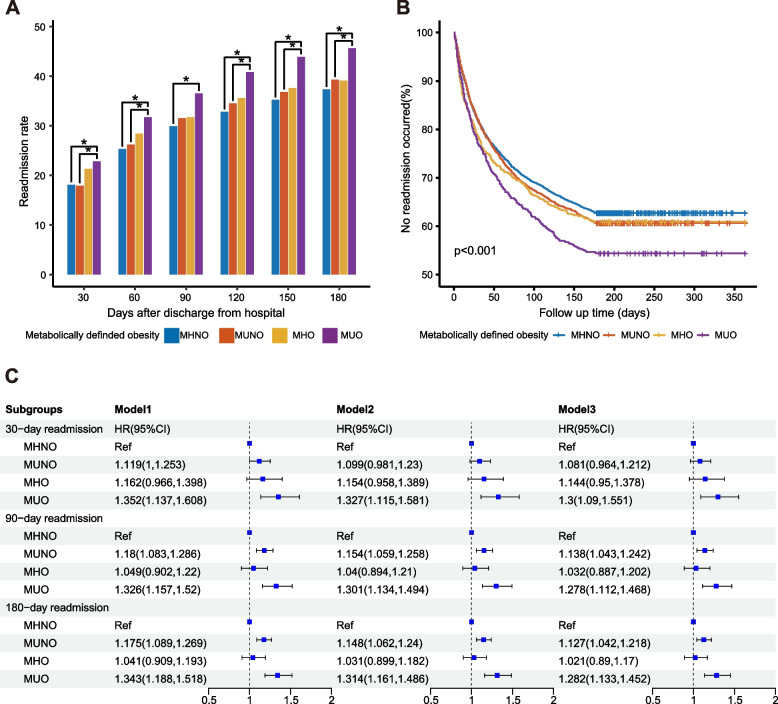
The association of obesity defined by metabolic status with readmission of female patients with NHL. **A** Readmission rates of female patients with NHL by the number of days following discharge from index hospitalization, stratified by metabolically defined obesity. The symbol * meant that the readmission rates of the two groups were significantly different at the 0.05 level after the Bonferroni correction. **B** Kaplan-Meier curve for readmission of female NHL patients, stratified by metabolically defined obesity. **C** COX regression was conducted to assess the association between metabolically defined obesity status and readmission of female patients with NHL. Factors adjusted in the COX regression: Model1: age, sex; Model2: Model1+ discharge disposition, discharge month, local hospital admission, household income, emergency department service, insurance type, and location of residence; Model3: Model2+ CCI. Abbreviations: MHNO, metabolically healthy non-obese; MUNO, metabolically unhealthy non-obese; MHO, metabolically healthy obese; MUO, metabolically unhealthy obese

The more significant impact of metabolic-defined obesity on readmission was observed in females than males. Figure 3 shows the association of metabolic-defined obesity with readmission among female NHL patients. Readmission rates were higher for MUO than for MHNO at all time points (*P* < 0.05). The survival curve showed that compared with MHNO, both MUNO and MHO had higher readmission rates, and the readmission rate increased further when obesity and metabolic abnormalities coexisted. Log-rank test (*P* < 0.001) showed that readmission rates in different groups were significantly different. COX regression indicated that MUNO and MUO were associated with increased risk of 90-day and 180-day readmission, while MHO was not. This suggested that metabolic abnormalities, rather than obesity, had an important impact on the increased risk of readmission in female patients. It was worth noting that the readmission rate and the HR value of readmission risk of MUO were higher than that of MUNO, suggesting that obesity may aggravate the association of metabolic abnormalities with readmission in female patients.

**Fig. 4:**
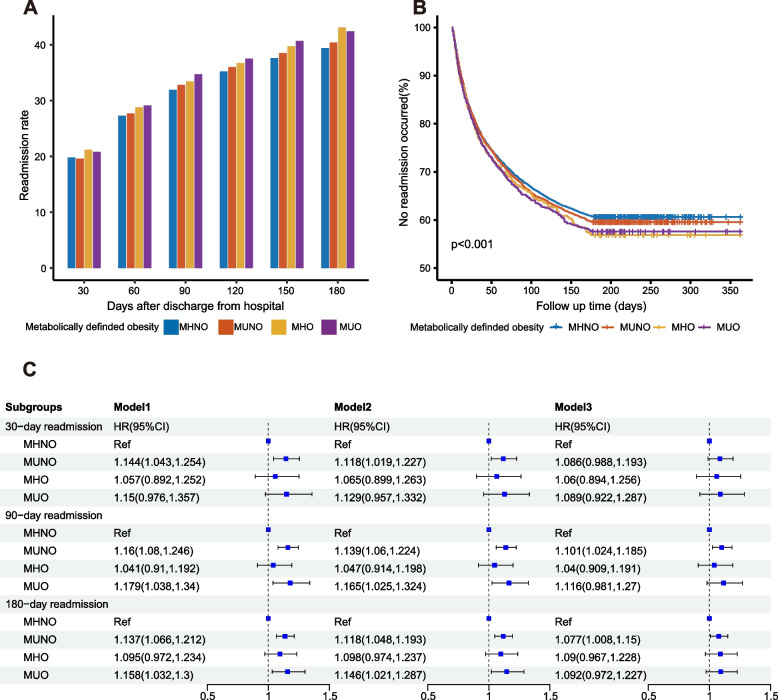
The association of obesity defined by metabolic status with readmission of male patients with NHL. **A** Readmission rates of male patients with NHL by the number of days following discharge from index hospitalization, stratified by metabolically defined obesity. The symbol * meant that the readmission rates of the two groups were significantly different at the 0.05 level after the Bonferroni correction. **B** Kaplan-Meier curve for readmission of male NHL patients, stratified by metabolically defined obesity. **C** COX regression was conducted to assess the association between metabolically defined obesity status and readmission of male patients with NHL. Factors adjusted in the COX regression: Model1: age, sex; Model2: Model1+ discharge disposition, discharge month, local hospital admission, household income, emergency department service, insurance type, and location of residence; Model3: Model2+ CCI. Abbreviations: MHNO, metabolically healthy non-obese; MUNO, metabolically unhealthy non-obese; MHO, metabolically healthy obese; MUO, metabolically unhealthy obese

In male patients, there was no significant difference in readmission rates among the groups (Fig. 4). COX regression indicated that only MUNO was associated with an elevated risk of readmission for 90 days and 180 days in Model 3, compared with MHNO. This suggested that metabolic-defined obesity may affect females more than males.

## Discussion

Based on this large national study of NHL inpatients in 2018, we characterized the association between metabolic-defined obesity and the risk of readmission of patients with NHL. MUO had higher readmission rates than MHNO when the time from discharge was 60 days or greater. Readmission rates of 150 and 180 days were higher in MUO than in MUNO. In the COX regression, MUNO and MUO were associated with a higher risk of 30-day, 90-day, and 180-day readmission, while MHO was not. Further classification showed that non-obese patients only with hyperglycemia, hypertension, or hyperlipidemia, and obesity only with hypertension had a higher risk of readmission than non-obese patients without metabolic abnormality. Furthermore, we found that an increase in the number of metabolic abnormalities was associated with a higher risk of readmission in non-obese patients. In addition, obesity combined with metabolic abnormalities had the strongest association with aggressive NHL. In our study, the association of metabolic-defined obesity with readmission was stronger for females than for males. To our knowledge, this was the first and largest study to assess the association of metabolic-defined obesity with readmission risk in patients with NHL.

Our analysis suggested that obesity alone had no association with the risk of readmission for NHL. However, metabolic abnormalities increased the risk of readmission significantly in patients with NHL, both in obese and non-obese patients. This may indicate the association of metabolic abnormalities with NHL. Brunello A reported that hyperglycemia during chemotherapy was associated with enhanced toxicity for hematologic tumors [26]. Furthermore, hyperglycemia often occurs in conjunction with hyperinsulinemia, chronic inflammation, and oxidative stress, which may accelerate tumor development in a variety of ways [27]. It is reported that preexisting hypertension increased the risk of all cardiovascular events after treatment in patients with aggressive NHL [19]. Hypertension was also proved to increase the cardiotoxicity of anthracycline [28]. The clinical association between blood lipids and the prognosis of NHL is understudied. Pugliese L et al. showed in vitro that as compared to normal when the concentration of cholesterol increased to 280 mg/dl, non-Hodgkin’s T cell human lymphoblastic lymphoma cells would incorporate cholesterol avidly for growth [29]. More studies are needed to elucidate the association between blood lipids and the prognosis of NHL. Considering the high prevalence and modifiability of metabolic abnormalities, it is necessary to pay attention to metabolic improvement in patients with NHL for a better prognosis, both in obese and non-obese patients. In addition, our results suggest that reducing the number of metabolic abnormality components, if possible, may benefit the prognosis of patients with NHL. Non-obese patients with three metabolic abnormalities may have a poorer prognosis and therefore require more attention.

Our study suggests that a stronger association between metabolic-defined obesity and readmission existed in female rather than male patients with NHL. In a study of elderly patients who had aggressive B cell lymphoma, the association between obesity and prognosis was statistically significant in female patients, while not in male patients [11]. Moreover, the prognosis of NHL is different between the sexes, in which male patients always have a worse prognosis [30]. Although the specific mechanisms behind the sex differences in the association of metabolic-defined obesity with readmissions of NHL patients remain unclear, our study may offer a reference for sex-stratified disease care and prognostic assessment to some extent.

Our study has the following advantages. For all we know, this is the first study taking into account the concomitant metabolic abnormalities to assess the real association of obesity with the readmission risk of NHL patients. We identified the limitations of BMI-defined obesity in the prognostic assessment of NHL patients and demonstrated the validity of metabolic-defined obesity. Besides, the NRD is a nationally representative database, containing data from all community hospitals of 28 states for all types of expected payers, which ensures sample size and representativeness [23]. Despite these advantages, our study was subject to several limitations. Because the NRD database records admissions for the same patient within a calendar year, and information for the same patient in different calendar years cannot be linked. We cannot track the ultimate outcome of NHL patients. Some factors affecting the prognosis of NHL were lacking, such as B symptoms, Eastern Cooperative Oncology Group performance status, Ann Arbor stage, and lactate dehydrogenase concentration. Medication use was also not available in the NRD. Besides, we did not have access to out-of-hospital mortality data which may lead to biased results. Both metabolic abnormalities and obesity categories were identified using the ICD-10 codes, further studies using specific laboratory measurement indicators are necessary to confirm the findings.

## Conclusions

The presence of metabolic abnormalities with or without obesity increased the risk of non-selective readmission in patients with non-Hodgkin’s lymphoma. However, obesity alone had no associations with the risk of non-selective readmission, suggesting that interventions for metabolic abnormalities may be more important in reducing the readmissions of patients with non-Hodgkin’s lymphoma.

### Supplementary Information

**Additional file 1: Text S1.** Description of confounding factors. **Text S2.** The Association of Obesity Defined by Metabolic Status with Readmission of Patients with Aggressive NHL. **Figure S1.** Flowchart of Cohort Selection. **Figure S2.** The Association of Obesity Defined by Metabolic Status with Readmission of Patients with Aggressive NHL. **Figure S3.** Readmission Rate by the Number of Days Following Discharge from Index Hospitalization. **Table S1.** Subtypes of Non-Hodgkin Lymphoma. **Table S2.** Diagnosis Codes, Used in Inclusion, Exclusion and Classification of Patients. **Table S3.** Main Diagnosis of Readmission. **Table S4.** Diagnosis Codes of Charlson Comorbidity Index. **Table S5.** Diagnosis Codes of Aggressive Non-Hodgkin Lymphoma.

## Data Availability

The data that support the findings of this study are available from the corresponding author upon reasonable request.

## Abbreviations

USA: United States of America
NHL: Non-Hodgkin lymphoma
NRD: Nationwide Readmissions Database
ICD-10: International Classification of Diseases, Tenth Edition
BMI: Body mass index
MHNO: Metabolically healthy non-obese
MUNO: Metabolically unhealthy non-obese
MHO: Metabolically healthy obese
MUO: Metabolically unhealthy obese
CCI: Charlson comorbidity index

## References

[1] Cai W, Zeng Q, Zhang X, Ruan W (2021). Trends analysis of non-Hodgkin lymphoma at the national, regional, and global level, 1990-2019: results from the global burden of disease study 2019. Front Med (Lausanne)..

[2] National Cancer Institute. Financial Burden of Cancer Care. https://progressreport.cancer.gov/after/economic_burden. Accessed 1 May 2022.

[3] Jencks SF, Williams MV, Coleman EA (2009). Rehospitalizations among patients in the Medicare fee-for-service program. N Engl J Med..

[4] Eskander RN, Chang J, Ziogas A, Anton-Culver H, Bristow RE (2014). Evaluation of 30-day hospital readmission after surgery for advanced-stage ovarian cancer in a medicare population. J Clin Oncol..

[5] Sullivan DH (1992). Risk factors for early hospital readmission in a select population of geriatric rehabilitation patients: the significance of nutritional status. J Am Geriatr Soc..

[6] Calle EE, Rodriguez C, Walker-Thurmond K, Thun MJ (2003). Overweight, obesity, and mortality from cancer in a prospectively studied cohort of U.S. adults. N Engl J Med..

[7] Gribsholt SB, Cronin-Fenton D, Veres K (2020). Hospital-diagnosed overweight and obesity related to cancer risk: a 40-year Danish cohort study. J Intern Med..

[8] Hales CM, Carroll MD, Fryar CD, Ogden CL (2017). Prevalence of Obesity Among Adults and Youth: United States, 2015-2016. NCHS Data Brief..

[9] Finkelstein EA, Khavjou OA, Thompson H (2012). Obesity and severe obesity forecasts through 2030. Am J Prev Med..

[10] Chihara D, Larson MC, Robinson DP (2021). Body mass index and survival of patients with lymphoma. Leuk Lymphoma..

[11] Hohloch K, Altmann B, Pfreundschuh M (2018). Obesity negatively impacts outcome in elderly female patients with aggressive B-cell lymphomas treated with R-CHOP: results from prospective trials of the German high grade non-Hodgkin's lymphoma trial group. Br J Haematol..

[12] Carson KR, Bartlett NL, McDonald JR (2012). Increased body mass index is associated with improved survival in United States veterans with diffuse large B-cell lymphoma. J Clin Oncol..

[13] Hong F, Habermann TM, Gordon LI (2014). The role of body mass index in survival outcome for lymphoma patients: US intergroup experience. Ann Oncol..

[14] Elagizi A, Kachur S, Lavie CJ (2018). An overview and update on obesity and the obesity paradox in cardiovascular diseases. Prog Cardiovasc Dis..

[15] Kammerlander AA, Mayrhofer T, Ferencik M (2021). Association of metabolic phenotypes with coronary artery disease and cardiovascular events in patients with stable chest pain. Diabetes Care..

[16] Hirode G, Wong RJ (2020). Trends in the prevalence of metabolic syndrome in the United States, 2011-2016. Jama..

[17] Debata A, Yoshida K, Ujifuku K (2017). Hyperglycemia is associated with poor survival in primary central nervous system lymphoma patients. Tumori..

[18] Drozd-Sokolowska J, Zaucha JM, Biecek P (2020). Type 2 diabetes mellitus compromises the survival of diffuse large B-cell lymphoma patients treated with (R)-CHOP - the PLRG report. Sci Rep..

[19] Moser EC, Noordijk EM, van Leeuwen FE (2006). Long-term risk of cardiovascular disease after treatment for aggressive non-Hodgkin lymphoma. Blood..

[20] Bluher M (2020). Metabolically Healthy Obesity. Endocr Rev..

[21] Hinnouho GM, Czernichow S, Dugravot A (2015). Metabolically healthy obesity and the risk of cardiovascular disease and type 2 diabetes: the Whitehall II cohort study. Eur Heart J..

[22] Hamer M, Batty GD, Kivimaki M (2012). Risk of future depression in people who are obese but metabolically healthy: the English longitudinal study of ageing. Mol Psychiatry..

[23] Healthcare Cost and Utilization Project. Introduction to the HCUP Nationwide Readmissions Database (NRD). https://hcup-us.ahrq.gov/db/nation/nrd/Introduction_NRD_2010-2018.jsp. Accessed 22 April 2022.

[24] Cheng Y, Han J, Li Q (2022). Metabolic obesity phenotypes: a friend or foe of digestive polyps?-An observational study based on National Inpatient Database. Metabolism..

[25] Brusselaers N, Lagergren J (2017). The Charlson Comorbidity Index in Registry-based Research. Methods Inf Med..

[26] Brunello A, Kapoor R, Extermann M (2011). Hyperglycemia during chemotherapy for hematologic and solid tumors is correlated with increased toxicity. Am J Clin Oncol..

[27] Ryu TY, Park J, Scherer PE (2014). Hyperglycemia as a risk factor for cancer progression. Diabetes Metab J..

[28] Qiu S, Zhou T, Qiu B (2021). Risk factors for anthracycline-induced cardiotoxicity. Front Cardiovasc Med..

[29] Pugliese L, Bernardini I, Pacifico N (2010). Severe hypocholesterolaemia is often neglected in haematological malignancies. Eur J Cancer..

[30] Cronin-Fenton DP, Sharp L, Deady S, Comber H (2006). Treatment and survival for non-Hodgkin's lymphoma: influence of histological subtype, age, and other factors in a population-based study (1999-2001). Eur J Cancer..

