# Supplementary material for: The classification of obesity based on metabolic status redefines the readmission of non-Hodgkin’s lymphoma—an observational study
Source: Cancer Metab. 2023 Dec 6;11:24. doi: 10.1186/s40170-023-00327-x (PMC10698918; doi:10.1186/s40170-023-00327-x)
Supplement: Supplementary file 1 — Additional file 1: Text S1. Description of confounding factors. Text S2. The Association of Obesity Defined by Metabolic Status with Readmission of Patients with Aggressive NHL. Figure S1. Flowchart of Cohort Selection. Figure S2. The Association of Obesity Defined by Metabolic Status with Readmission of Patients with Aggressive NHL. Figure S3. Readmission Rate by the Number of Days Following Discharge from Index Hospitalization. Table S1. Subtypes of Non-Hodgkin Lymphoma. Table S2. Diagnosis Codes, Used in Inclusion, Exclusion and Classification of Patients. Table S3. Main Diagnosis of Readmission. Table S4. Diagnosis Codes of Charlson Comorbidity Index. Table S5. Diagnosis Codes of Aggressive Non-Hodgkin Lymphoma. [file 40170_2023_327_MOESM1_ESM.pdf]

# Supplementary Materials

## The Classification of Obesity Based on Metabolic Status Redefines the Readmission of non-Hodgkin's Lymphoma


**Authorship:**

Hang Dong, Honglin Guo, Jing Du, Yiping Cheng, Dawei Wang, Junming Han, Zinuo Yuan, Zhenyu Yao, Ran An, Xiaoqin Wu, Kyle L Poulsen, Zhixiang Wang, Shanshan Shao, Xiude Fan#, Zhen Wang#, Jiajun Zhao#

**#Corresponding Authors:**

Jiajun Zhao, jjzhao@sdu.edu.cn.
Xiude Fan, fanxiudexjtu@163.com.
Zhen Wang, wangzhen@sdfmu.edu.cn.


## Contents


Text S1: Description of confounding factors ........ 2
Text S2: The Association of Obesity Defined by Metabolic Status with Readmission of Patients with Aggressive NHL. ........ 3
Figure S1: Flowchart of Cohort Selection. ........ 4
Figure S2: The Association of Obesity Defined by Metabolic Status with Readmission of Patients with Aggressive NHL. ........ 5
Figure S3 Readmission Rate by the Number of Days Following Discharge from Index Hospitalization. ........ 7
Table S1: Subtypes of Non-Hodgkin Lymphoma. ........ 8
Table S2: Diagnosis Codes, Used in Inclusion, Exclusion and Classification of Patients. ........ 10
Table S3: Main Diagnosis of Readmission. ........ 11
Table S4: Diagnosis Codes of Charlson Comorbidity Index. ........ 12
Table S5: Diagnosis Codes of Aggressive Non-Hodgkin Lymphoma. ........ 13
References ........ 14

**Text S1: Description of confounding factors**

In order to adjust the impact of confounding factors on the results, we further included variables related to hospitalization in the COX regression, including discharge disposition, discharge month, local hospital admission, household income, emergency department service, insurance type, and location of residence. Discharge disposition represents the disposition of the patient at discharge, including routine, transfer to another hospital, home health, etc. Patients who died at first hospitalization were excluded, and not included in the analysis. Local hospital admission reflects whether patients seek medical care locally or at hospitals in other states. Hospitals that specialize in a certain type of care may attract patients from all over the United States. Patients with more severe or complex conditions may also be more inclined to travel to hospitals farther away in search of better treatment. Household income were categorized into a quartile classification based on household income median in zip code of patients. Emergency department service indicated whether the patient has evidence of receiving emergency medical services while hospitalized.[1] Location of patients was categorized by urban-rural classification scheme formulated by the National Center for Health Statistics.[2]

**Text S2: The Association of Obesity Defined by Metabolic Status with Readmission of Patients with Aggressive NHL.**

Aggressive NHL tend to progress more rapidly and require more treatment and intervention, so we conducted a further analysis of readmission rates for aggressive NHL. For patients with aggressive NHL, there was no statistically significant difference in readmission rates between MUNO MHO, and MHNO (Figure S2). But there was a statistically significant increase in readmission rates for MUO, when the time of discharge from hospital was 60 days or more. These results suggested that the combined presence of obesity and metabolic abnormalities had a greater adverse effect on readmission than obesity alone and metabolic abnormalities alone. The survival curve indicated that readmission rates were slightly elevated for MUNO and MHO compared with MHNO, whereas greatly elevated for MUO (Figure S2.B). The result of the log-rank test was statistically significant (P<0.001). After adjustment for sex and age, MUNO was associated with elevated risk of 90-day readmission (HR=1.105, 95% CI: 1.011-1.208), while MUO was associated with higher risk of 30-day (HR=1.362, 95% CI: 1.137-1.633), 90-day (HR=1.423, 95% CI: 1.235-1.639) and 180-day readmission risk (HR=1.408, 95% CI: 1.236-1.604) (Figure S2.C). The results were similar for MUO in Model2 and Model3.

In this study, as a modifiable factor, metabolic ill-health had a negative effect on readmission for aggressive NHL, especially when comorbid with obesity. This suggested that for patients with aggressive NHL, although obesity alone had no significant effect on readmission, obesity may exacerbate the adverse effect of metabolic abnormalities on readmission. Current studies showed that obesity could lead to the increase of leptin, IL-6, IGF and insulin, and then lead to the activation of JAK/STAT, MAPK, PI3K and other signaling pathways, which further promoted cell growth and proliferation and inhibited cell apoptosis, which may be one of the reasons for the adverse effect of obesity on aggressive NHL.[3] Therefore, for patients with NHL, especially those with aggressive NHL, it maybe still beneficial to maintain normal body weight while improving metabolic abnormalities for improving prognosis.

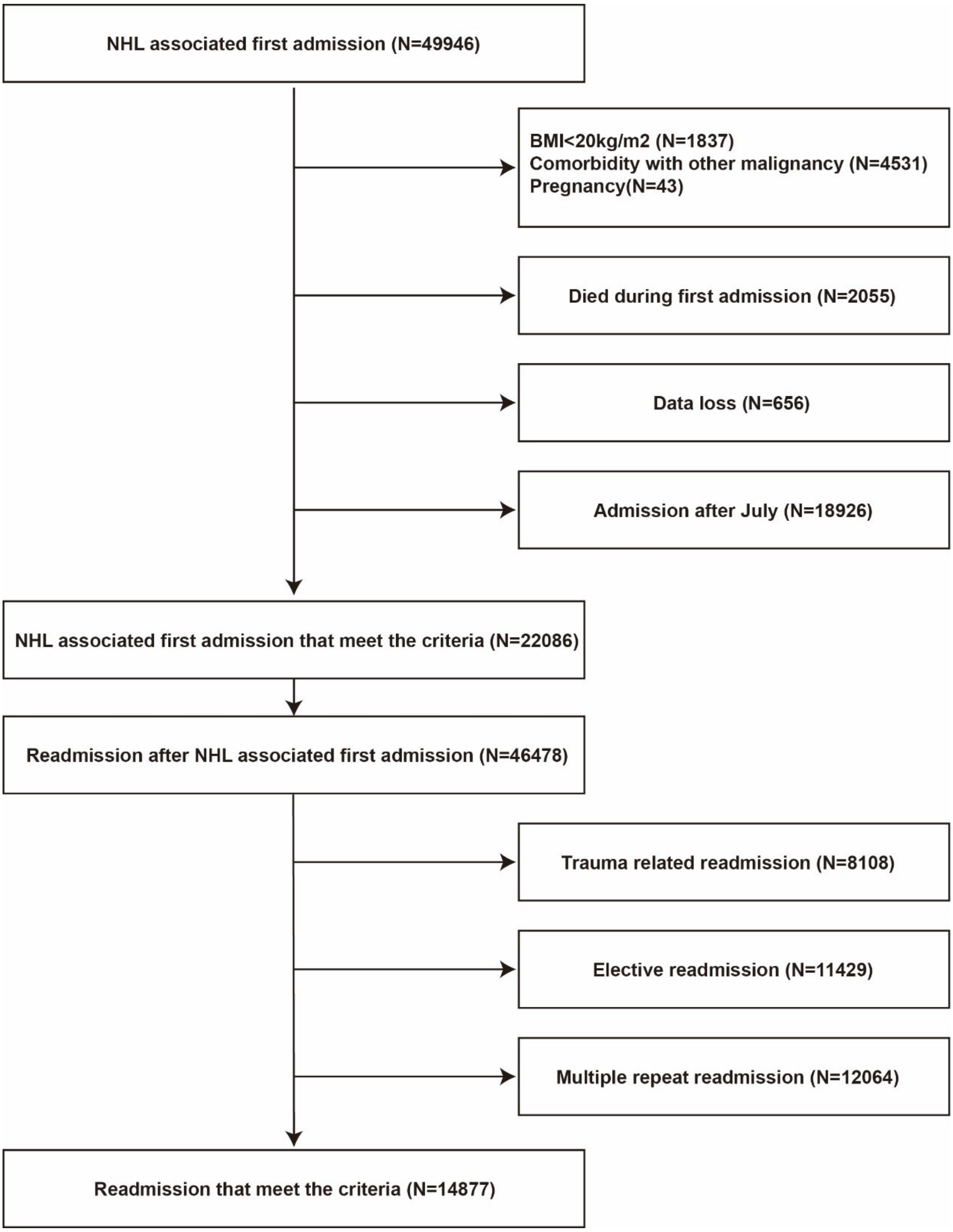


**Figure S1: Flowchart of Cohort Selection.**

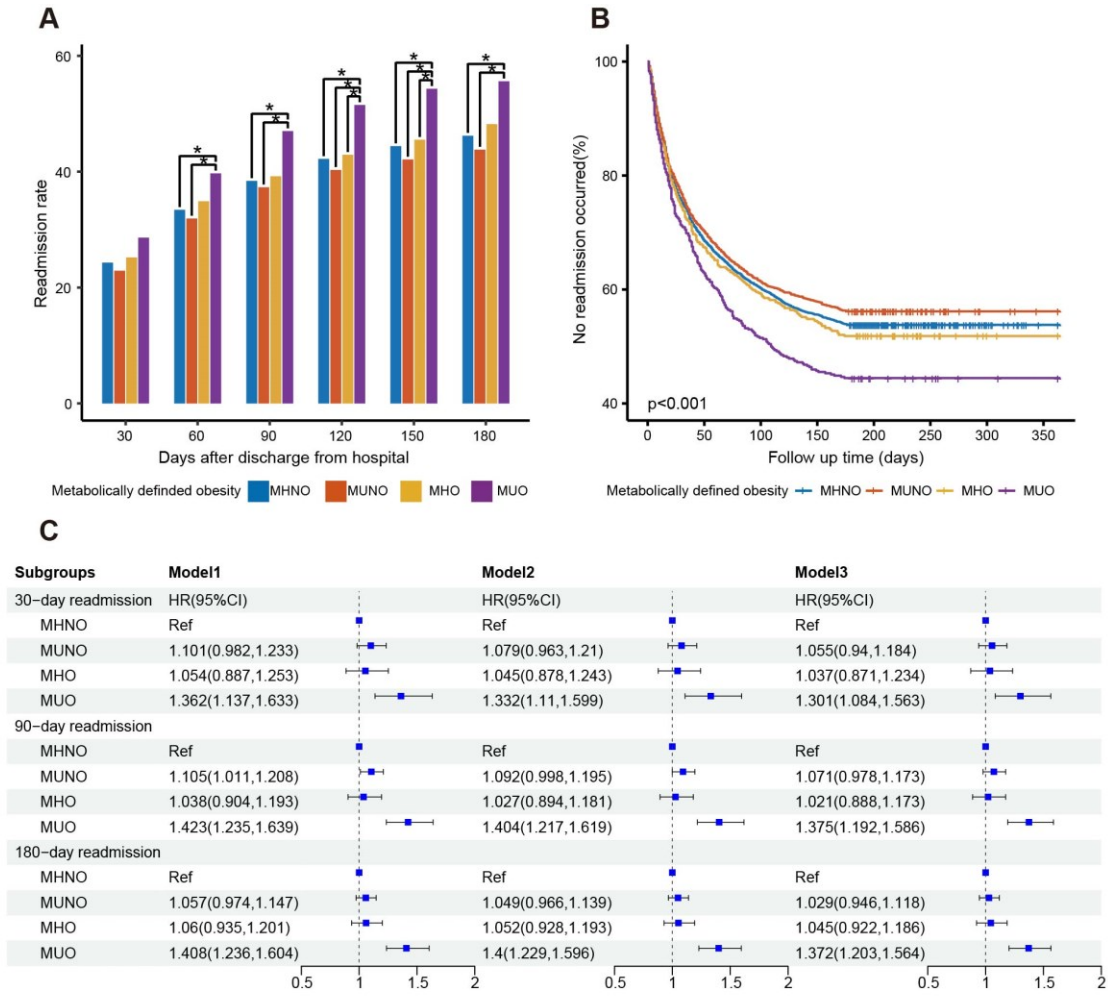


**Figure S2: The Association of Obesity Defined by Metabolic Status with Readmission of Patients with Aggressive NHL.**

(A) Readmission rates of patients with aggressive NHL by the number of days following discharge from index hospitalization, stratified by metabolically defined obesity. The symbol * meant that the readmission rates of the two groups were significantly different at the 0.05 level after the Bonferroni correction. (B) Kaplan-Meier curve for readmission of aggressive NHL patients, stratified by metabolically defined obesity. (C) COX regression was conducted to assess the association between metabolically defined obesity status and readmission of patients with aggressive NHL. Factors adjusted in the COX regression: Model1: age, sex; Model2: Model1+ discharge disposition, discharge month, local hospital admission, household income, emergency department service, insurance type, and location of residence; Model3: Model2+ CCI.

Abbreviations: HR, hazard ratio; CI, confidence interval; MHNO, metabolically healthy non-obese; MUNO, metabolically unhealthy non-obese; MHO, metabolically healthy obese; MUO, metabolically

unhealthy obese.

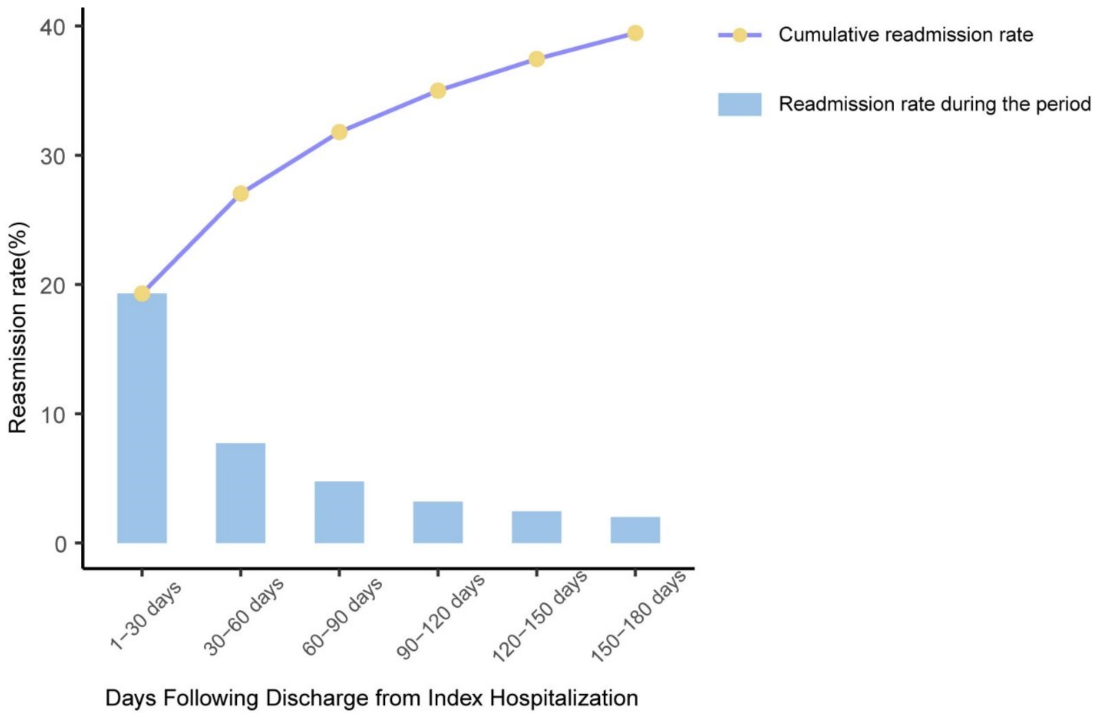


**Figure S3 Readmission Rate by the Number of Days Following Discharge from Index Hospitalization.**

**Table S1: Subtypes of Non-Hodgkin Lymphoma.**

| **Subtypes of Non-Hodgkin Lymphoma** | **ICD-10 codes** | **Number** | **Percent(%)** |
|---|---|---|---|
| Follicular lymphoma | C820-C824 | 472 | 2.14 |
| Diffuse follicle center lymphoma | C825 | 25 | 0.11 |
| Cutaneous follicle center lymphoma | C826 | 3 | 0.01 |
| Other types of follicular lymphoma | C828 | 87 | 0.39 |
| Follicular lymphoma, unspecified | C829 | 643 | 2.91 |
| Small cell B-cell lymphoma | C830 | 301 | 1.36 |
| Mantle cell lymphoma | C831 | 931 | 4.22 |
| Diffuse large B-cell lymphoma | C833 | 5088 | 23.04 |
| Lymphoblastic (diffuse) lymphoma | C835 | 183 | 0.83 |
| Burkitt lymphoma | C837 | 332 | 1.5 |
| Other non-follicular lymphoma | C838 | 44 | 0.2 |
| Non-follicular (diffuse) lymphoma, unspecified | C839 | 5 | 0.02 |
| Mycosis fungoides | C840 | 119 | 0.54 |
| Sezary disease | C841 | 41 | 0.19 |
| Peripheral T-cell lymphoma | C844 | 259 | 1.17 |
| Anaplastic large cell lymphoma, ALK-positive | C846 | 64 | 0.29 |
| Anaplastic large cell lymphoma, ALK-negative | C847 | 70 | 0.32 |
| Mature T/NK-cell lymphomas, unspecified | C849 | 20 | 0.09 |
| Cutaneous T-cell lymphoma | C84A | 199 | 0.9 |
| Other mature T/NK-cell lymphomas | C84Z | 9 | 0.04 |
| Unspecified B-cell lymphoma | C851 | 1700 | 7.7 |
| Mediastinal (thymic) large B-cell lymphoma | C852 | 204 | 0.92 |
| Other specified types of non-Hodgkin lymphoma | C858 | 520 | 2.35 |
| Non-Hodgkin lymphoma, unspecified | C859 | 3745 | 16.96 |
| Extranodal NK/T-cell lymphoma, nasal type | C860 | 21 | 0.1 |
| Hepatosplenic T-cell lymphoma | C861 | 18 | 0.08 |
| Enteropathy-type (intestinal) T-cell lymphoma | C862 | 12 | 0.05 |
| Subcutaneous panniculitis-like T-cell lymphoma | C863 | 12 | 0.05 |
| Blastic NK-cell lymphoma | C864 | 26 | 0.12 |
| Angioimmunoblastic T-cell lymphoma | C865 | 145 | 0.66 |
| Primary cutaneous CD30-positive T-cell proliferations | C866 | 29 | 0.13 |
| Waldenstrom macroglobulinemia | C880 | 489 | 2.21 |
| Heavy chain disease | C882 | 7 | 0.03 |
| Immunoproliferative small intestinal disease | C883 | 0 | 0 |
| Extranodal marginal zone B-cell lymphoma of mucosa-associated lymphoid tissue [MALT-lymphoma] | C884 | 244 | 1.1 |
| Other malignant immunoproliferative diseases | C888 | 1 | 0 |
| Malignant immunoproliferative disease, unspecified | C889 | 1 | 0 |

| | | | |
|---|---|---|---|
| Chronic lymphocytic leukemia of B-cell type | C911 | 5638 | 25.53 |
| Prolymphocytic leukemia of B-cell type | C913 | 12 | 0.05 |
| Hairy cell leukemia | C914 | 211 | 0.96 |
| Adult T-cell lymphoma/leukemia (HTLV-1-associated) | C915 | 116 | 0.53 |
| Prolymphocytic leukemia of T-cell type | C916 | 40 | 0.18 |
| Mature B-cell leukemia Burkitt-type | C91A | 9 | 0.04 |
| Malignant neoplasm of lymphoid, hematopoietic and related tissue, unspecified | C969 | 101 | 0.46 |
| Other specified malignant neoplasms of lymphoid, hematopoietic and related tissue | C96Z | 0 | 0 |
| Post-transplant lymphoproliferative disorder (PTLD) | D47Z1 | 153 | 0.69 |

**Table S2: Diagnosis Codes, Used in Inclusion, Exclusion and Classification of Patients.**

| **Diagnoses** | **ICD-10 Codes** |
|---|---|
| Non-Hodgkin lymphoma | C82-86, C88, C911, C913-916, C91A, C969, C96Z. D47Z1 |
| Underweight | Z681 |
| Other malignant tumors | C00–26, C30–34, C37–41, C43, C45–58, C60–76, C80–81,C90, C919, C91Z,C92-966, C96A,C96Z |
| Pregnancy | Z31, Z34, Z36, Z3A, O00-O02 |
| Obesity | Z6825-6845, E6601, E662-663 |
| Hyperlipidemia | E780-785 |
| Hypertension | G932, H35031, H35032, H35033, H35039, I10-13, I15, I16, I674, I973, R030 |
| Hyperglycemia | E08, E10, E11, E13, R7301, R7302, R7303 |
| Trauma | V00-Y99 |

**Table S3: Main Diagnosis of Readmission.**

| Primary Diagnosis of Readmission | ICD-10 codes | 30-day Readmission N(%) | 60-day Readmission N(%) | 90-day Readmission N(%) |
|---|---|---|---|---|
| **Infectious and parasitic diseases** | A00-B99 | 326(7.65) | 545(7.76) | 690(7.92) |
| **Tumors** | C00-D48 | 1920(45.04) | 3037(43.25) | 3589(41.2) |
| Non-Hodgkin's lymphoma | | 1896(44.48) | 2999(42.71) | 3539(40.62) |
| Other tumors | | 24(0.56) | 38(0.54) | 50(0.57) |
| **Diseases of the blood, hematopoietic organs and certain abnormalities involving immune function** | D50-D89 | 170(3.99) | 274(3.9) | 339(3.89) |
| **Endocrine, nutritional and metabolic diseases** | E00-E90 | 70(1.64) | 133(1.89) | 182(2.09) |
| **Mental and behavioral disorders** | F00-F99 | 21(0.49) | 37(0.53) | 50(0.57) |
| **Nervous system diseases** | G00-G99 | 36(0.84) | 61(0.87) | 89(1.02) |
| **Eye and accessory diseases/ ear and mastoid diseases** | H00-H95 | 1(0.02) | 4(0.06) | 4(0.05) |
| **Circulatory system diseases** | I00-I99 | 301(7.06) | 574(8.17) | 742(8.52) |
| **Respiratory diseases** | J00-J99 | 307(7.2) | 522(7.43) | 694(7.97) |
| **Digestive diseases** | K00-K93 | 174(4.08) | 286(4.07) | 398(4.57) |
| **Diseases of skin and subcutaneous tissue** | L00-L99 | 49(1.15) | 97(1.38) | 129(1.48) |
| **Musculoskeletal system and connective tissue disorders** | M00-M99 | 51(1.2) | 83(1.18) | 117(1.34) |
| **Diseases of genitourinary system** | N00-N99 | 104(2.44) | 194(2.76) | 262(3.01) |
| **Pregnancy, childbirth and puerperium** | O00-O99 | | 1(0.01) | 1(0.01) |
| **Symptoms, signs and abnormal clinical and laboratory results** | R00-R99 | 78(1.83) | 135(1.92) | 178(2.04) |
| **Injury, poisoning and other results of external causes** | S00-T98 | 98(2.3) | 195(2.78) | 259(2.97) |
| **Factors affecting health status and access to health services** | Z00-Z99 | 557(13.07) | 844(12.02) | 989(11.35) |
| **All** | | 4263(100) | 7022(100) | 8712(100) |

**Table S4: Diagnosis Codes of Charlson Comorbidity Index.**

| **Comorbidity** | **ICD-10** |
|---|---|
| Myocardial infarction | I22-I23, I252 |
| Congestive heart failure | I11, I13, I255, I42–43, I50, I517 |
| Peripheral vascular disease | I70–73, I770-I771, K551, K558–559, Z958–959 |
| Cerebrovascular disease | G45–46, I60–69 |
| Dementia | A810, F01–03, F051, G30–31 |
| Chronic pulmonary disease | I26–27, J40-J47, J60–67, J684, J701, J703 |
| Rheumatic disease | M05–06, M120, M315, M32-M36 |
| Liver disease | B18, I85, I864, I982, K70–71, K721, K729, K76, R162, Z944 |
| Diabetes mellitus | E10–13 |
| Hemiplegia/paraplegia | G114, G81–83 |
| Renal disease | I12–13, N01, N03, N05, N07, N08, N171, N172, N18, N19, N25, Z49, Z940, Z992 |
| Metastatic tumors | C77–79 |
| AIDS/HIV | B20 |

**Table S5: Diagnosis Codes of Aggressive Non-Hodgkin Lymphoma.**

| **Aggressive NHL** | **ICD-10 Codes** |
|---|---|
| Mantle cell lymphoma | C831 |
| Diffuse large B-cell lymphoma | C833 |
| Burkitt lymphoma | C837 |
| Lymphoblastic (diffuse) lymphoma | C835 |
| Sezary disease | C841 |
| Peripheral T-cell lymphoma | C844 |
| Anaplastic large cell lymphoma, ALK-positive | C846 |
| Anaplastic large cell lymphoma, ALK-negative | C847 |
| Mediastinal (thymic) large B-cell lymphoma | C852 |
| Extranodal NK/T-cell lymphoma, nasal type | C860 |
| Hepatosplenic T-cell lymphoma | C861 |
| Enteropathy-type (intestinal) T-cell lymphoma | C862 |
| Blastic NK-cell lymphoma | C864 |
| Angioimmunoblastic T-cell lymphoma | C865 |
| Primary cutaneous CD30-positive T-cell proliferations | C866 |
| Prolymphocytic leukemia of B-cell type | C913 |
| Adult T-cell lymphoma/leukemia (HTLV-1-associated) | C915 |
| Prolymphocytic leukemia of T-cell type | C916 |
| Mature B-cell leukemia Burkitt-type | C91A |
| Post-transplant lymphoproliferative disorder (PTLD) | D47Z1 |

**References**

1 Healthcare Cost and Utilization Project. Introduction to the HCUP Nationwide Readmissions Database (NRD). https://hcup-us.ahrq.gov/db/nation/nrd/Introduction_NRD_2010-2018.jsp. Accessed 22 April 2022.

2 Centers for Disease Control and Prevention, National Center for Health Statistics. NCHS urban–rural classification scheme for counties. https://www.cdc.gov/nchs/data_access/urban_rural.htm. Accessed 27 April 2022.

3 Hopkins BD, Goncalves MD, Cantley LC. Obesity and cancer mechanisms: cancer metabolism. *J Clin Oncol* 2016; **34(35)**: 4277-4283.
